# Posterior Tibial Nerve Stimulation for the Treatment of Detrusor Overactivity in Multiple Sclerosis Patients: A Narrative Review

**DOI:** 10.3390/jpm14040355

**Published:** 2024-03-28

**Authors:** Vaia Sapouna, Athanasios Zikopoulos, Sofia Thanopoulou, Dimitrios Zachariou, Ioannis Giannakis, Aris Kaltsas, Bou Sopheap, Nikolaos Sofikitis, Athanasios Zachariou

**Affiliations:** 1 Department of Urology, EV PRATTEIN Rehabilitation Centre, 38222 Volos, Greece; sthanopoulou@uth.gr (S.T.); dzachar@med.uoa.gr (D.Z.); 2 Department of Urology, Faculty of Medicine, School of Health Sciences, University of Ioannina, 45110 Ioannina, Greece; athanasios.zikopoulos1@nhs.net (A.Z.); iogiannak@cc.uoi.gr (I.G.); nsofikit@uoi.gr (N.S.); azachariou@uoi.gr (A.Z.); 3 Third Department of Urology, Attikon University Hospital, School of Medicine, National and Kapodistrian University of Athens, 12462 Athens, Greece; a.kaltsas@uoi.gr; 4 Department of Urology, Cambodia-China Friendship Preah Kossamak Hospital, Phnom Penh 120406, Cambodia; bousopheap111@gmail.com

**Keywords:** bladder dysfunction, multiple sclerosis, neurogenic detrusor overactivity, non-invasive treatment, posterior tibial nerve stimulation and quality of life

## Abstract

Bladder dysfunction, particularly neurogenic detrusor overactivity (DO), poses a substantial challenge in multiple sclerosis (MS) patients, detrimentally impacting their quality of life (QoL). Conventional therapies often fall short, necessitating alternative approaches like posterior tibial nerve stimulation (PTNS) for effective management. This narrative review critically examines the application of PTNS in treating DO among MS patients, aiming to provide a comprehensive synthesis of its efficacy, underlying mechanisms, and clinical outcomes. By evaluating a spectrum of studies, including randomized controlled trials and long-term follow-up research, the review elucidates PTNS’s role in enhancing bladder control and ameliorating symptoms of urgency and incontinence, thereby improving patient well-being. Despite its potential, the review acknowledges the limited scope of existing research specific to MS-induced neurogenic DO and calls for further investigation to optimize PTNS protocols and understand its long-term benefits. Highlighting PTNS’s minimal invasiveness and favorable safety profile, the review advocates for its consideration as a viable third-line treatment option in MS-related bladder dysfunction management. Through this analysis, the review contributes to the broader narrative of seeking effective, patient-centered therapeutic strategies for MS-related complications, underscoring the importance of personalized care in improving patient outcomes.

## 1. Introduction

Multiple sclerosis (MS) is a chronic autoimmune disease characterized by inflammation, demyelination, and neurodegeneration within the central nervous system (CNS). This condition often presents with a wide array of symptoms, including bladder dysfunction, which can significantly impact the quality of life (QoL) for individuals affected [1].

The origins of MS remain uncertain, but it is commonly regarded as a multifactorial disorder involving a combination of genetic predisposition and environmental factors [2]. The immunological aspects of the disease underscore the importance of understanding the intricate interplay between the immune system and the CNS in the context of MS [3].

Bladder dysfunction such as neurogenic detrusor overactivity (DO) or detrusor sphincter dyssynergia (DSD) is a prevalent and impactful symptom complex in individuals with MS, significantly compromising their QoL. The complex neural regulation of the bladder is disturbed when lesions occur in the brain or spinal cord, disrupting nerve signals that control the detrusor muscle and urinary sphincter [4]. When dysfunctions occur in the lower urinary tract system, various symptoms arise, including increased frequency of urination, urgency, urge incontinence, and urinary leakage [5].

Traditional treatment approaches, including behavioral therapies and medications, may not always provide sufficient relief. In cases where patients are unresponsive to pharmacological treatment for neurogenic DO or experience intolerable side effects, alternative options such as repeated intramuscular injections of botulinum toxin into the detrusor or sacral modulation may be contemplated. If desired outcomes cannot be achieved through other means, surgical intervention may be considered. Given the evolving nature of the neurological symptoms associated with MS alongside urological disorders, a preference exists for non-destructive treatment options to mitigate or delay the need for surgery. This has led to the exploration of alternative interventions, such as posterior tibial nerve stimulation (PTNS), as a potential avenue for addressing bladder rehabilitation in individuals with MS [6]. PTNS involves the use of electrical impulses to modulate the neural pathways associated with bladder function and is a third-line treatment for refractory overactive bladder (OAB) treatment [5].

Given the diverse clinical presentations of MS, ranging from relapsing–remitting to progressive forms, comprehending the potential benefits and limitations of PTNS becomes paramount [5]. Through synthesizing and analyzing pertinent studies, this narrative review endeavors to provide valuable insights into the role of this innovative therapeutic approach in managing detrusor overactivity and consequently enhancing the overall well-being of individuals with MS. The review aims to offer significant insights into the function of PTNS in improving bladder function and subsequently by synthesizing and evaluating relevant literature to assess patient outcomes, technique, and general effects of PTNS on bladder function in the context of MS.

## 2. Multiple Sclerosis and Bladder Function

The micturition cycle involves a combination of reflex actions and conscious control [7]. Positioned within the frontal cortex, the cortical micturition control center regulates the filling phase. Its role includes inhibiting spinal reflexes and detrusor contractility to delay bladder voiding until optimal conditions are met. Signals from the cortical cortex are processed in intermediate sites, namely the pons and sacral spinal cord. Within the pons, the pontine micturition center and locus coeruleus coordinate sphincter relaxation and detrusor contraction, ensuring their synchronized function. Specifically, during filling phases, the sphincter is contracted and the detrusor relaxed, while the opposite occurs during the voiding phases. Additionally, the pons receives signals from bladder tensoreceptors, providing feedback to conscious centers regarding the urge to void [8].

In conditions such as MS, demyelination of these anatomical structures—cerebrum, brainstem, and spinal cord—can occur, leading to various urological symptoms due to diverse neurological damage patterns. Close to 90% of MS patients exhibit intracranial lesions distributed extensively throughout the white matter. Lesions in cortical regions correlated with micturition function (medial prefrontal cortex, insula, and pons) cause the most frequent dysfunction: diminished cortical control over detrusor reflexes (>60% patients) [9]. This leads to neurogenic DO, characterized by urinary urgency, frequency, nocturia, and urge incontinence. Spinal cord lesions, particularly those above the sacral region, are frequently observed in individuals with MS. The prevalence of cervical cord plaques approaches nearly 80% in such cases, mainly affecting the lateral corticospinal (pyramidal) and reticulospinal tracts. Involvement of the lumbar and dorsal cord occurs less frequently, with rates of around 40% and 18%, respectively. Suprasacral spinal lesions can induce neurogenic DO by interfering with the descending inhibition of bladder contraction. Spinal cord lesions damaging the reticulospinal tracts cause impaired bladder contractility, resulting in incomplete bladder emptying due to a lack of coordination between the pontine micturition center and the bladder, leading to bladder sphincter dyssynergia (about 25% of MS patients). Finally, 10–15% of patients have reduced sensation, leading to issues like urinary retention or incomplete bladder voiding. This condition arises from demyelination affecting sensory pathways that transmit bladder distention and volume signals to CNS [10].

Micturition issues in MS can stem from additional factors and may be associated with cognitive impairments, decreased mobility, lower urinary tract pathology (bladder neck obstruction, benign prostatic hyperplasia and urinary tract infections), or functional incontinence [11]. For instance, the presence of nocturia in MS patients might be connected to nocturnal polyuria. Demyelinating lesions in the spinal cord cause autonomic cardiovascular dysfunction or antidiuretic hormone (ADH) diurnal release alterations, resulting in nocturnal hypertension and nocturnal polyuria [12]. Additionally, MS patients often experience more sleep disturbances compared to healthy individuals. Sleep deprivation, which can disrupt circadian rhythms, may lead to increased nighttime urine production [13,14].

While only 3–10% of MS patients experience urological symptoms as their initial symptom, 50–90% of MS patients exhibit moderate-to-severe urine abnormalities at some time in their life. Urinary leakage is common, is positively connected with overall neurological status, and negatively influences patients’ QoL. Numerous studies have highlighted that MS patients with urinary disorders often experience severe morbidity and consequently should represent a priority for health-care professionals [15].

Different therapeutic options have been proposed to treat neurogenic DO in MS patients. Conservative treatments, including pelvic floor muscle training, bladder rehabilitation programs or toilet training, can partially improve patient symptoms and QoL [16]. Pharmacological options (anticholinergics, b3 adrenoreceptors, and intravesical injections of botulinum toxin) or other non-pharmacological treatments (self-intermittent catheterization and surgical procedures) can lead to side effects and thus poor adherence. Furthermore, in certain instances, individuals with MS may find themselves unable to carry out certain interventions, such as self-intermittent catheterization, due to the presence of motor, sensory, visual, or cognitive impairments [17].

For those who do not respond to these interventions, the use of tibial nerve stimulation has been proposed, supported by varying degrees of evidence [18]. This highlights the significance of neuromodulation techniques as a valuable strategy for enhancing neurogenic DO in MS patients.

## 3. Methods

A literature search was conducted on three databases (Medline through PubMed, Web of Science and Scopus). Targeted keywords, such as multiple sclerosis, bladder dysfunction, PTNS and neurogenic detrusor overactivity were deployed. Additionally, a meticulous scrutiny of reference lists from related articles was undertaken, reinforcing the commitment to a comprehensive exploration of the existing literature.

Inclusion criteria focused on studies involving adults with bladder disorders related to multiple sclerosis (MS). Studies that provided detailed descriptions of specific rehabilitation treatments were specifically sought. Furthermore, we included only research designs that were carefully chosen, including published prospective and retrospective studies as well as randomized controlled trials (RCTs). It was imperative that studies met these criteria for inclusion in our analysis. Furthermore, the language criterion was restricted to original English text to maintain consistency in data interpretation.

To refine our focus, we implemented specific exclusion criteria. Studies related to neurological diseases other than MS were excluded, as the emphasis of this narrative review was on the unique challenges presented by MS-related bladder disorders. Studies that did not explicitly address urinary symptoms were also excluded from our analysis. Furthermore, to eliminate redundancy and maintain the integrity of our findings, duplicate studies were meticulously excluded from consideration.

## 4. Posterior Tibial Nerve Simulation

The PTNS procedure was introduced by McGuire et al. in a group of incontinent patients [19]. Originally, it was used a transcutaneous electrode over the common peroneal or posterior tibial nerve and a ground electrode placed over the same nerve contralaterally. Later on, Stoller et al. presented a modification of the original method, which was FDA (Food and Drug Administration)-approved in 2000 [20].

**Figure 1:**
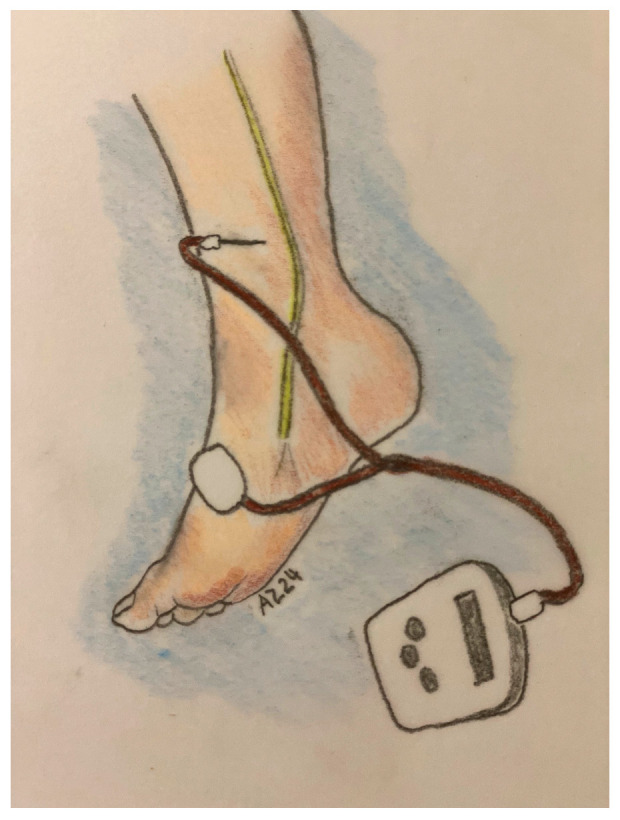
PTNS technique, Stoller modification.

The usual treatment protocol consists of 12 sessions conducted in a 6-week period. Each PTNS procedure lasts 30 min in Stoller’s modified process. The patients are instructed to lie down and bend their leg, while a needle is inserted 5 cm cephalad and 1.5 cm behind the medial malleolus, between the posterior margin of the tibia and the soleus muscle [5]. A transcutaneous adhesive contact electrode is placed posteriorly to the medial malleolus on the sole of the foot on the same leg. A low-voltage (9 V) electrical stimulator is connected to both the adhesive electrode and the needle (Figure 1) [21]. Utilizing a fixed frequency of 20 Hz and a pulse width of 200 ms, the stimulation current (ranging from 0 to 10 mA) is gradually increased until a motor response concurs with either the big toe’s plantar flexion or the “fan-shaped” opening of all toes. Throughout the session, the health-care professional adjusts the current intensity based on the patient’s tolerance level toward a tingling sense originating from the stimulation zone and radiating to the sole of the foot. If a clear motor response is not elicited, the insertion process should be repeated and the needle repositioned. In most cases, the motor response is accompanied by a light tingling sensation in the sole of the foot.

PTNS is a minimally invasive neuromodulation technique that has demonstrated efficacy in treating patients with neurogenic DO unresponsive to conventional medical therapy [21]. Despite its proven effectiveness, the precise mechanism underlying PTNS remains incompletely understood. Long-latency somatosensory evoked potentials (LL-SEPs) are widely recognized as indicators of brain information processing following peripheral somatosensory system stimulation. In a study by Finazzi-Agro et al. (2009) [22], LL-SEPs were assessed before and after PTNS treatment, revealing alterations in brain activity following PTNS. It is possible that PTNS effectiveness is facilitated through sacral and suprasacral centers involved in stimulus processing, potentially engaging relevant brain cortical regions [22]. PTNS is a neuromodulation method of the posterior tibial nerve, which contains fibers from the L4–S3 spinal levels. While the exact mechanism remains ambiguous, Caldwell [23] proposed that electrical stimulation of the tibial nerve might suppress detrusor activity by depolarizing somatic sacral and lumbar afferent fibers. These depolarized fibers probably inhibit preganglionic bladder motor neurons in the spinal cord [24]. Danisman et al. reported that the mast cell population in the bladder of female rats reduced following PTNS treatment [25]. Furthermore, PTNS treatment affects spinal cord function by reducing C-fos expression (an indicator of neuronal metabolic action) in the rat sacral spinal cord after electrical stimulation of the hind leg (Table 1) [26].

## 5. PTNS Is an Effective Treatment for Detrusor Overactivity in MS Patients

### 5.1. PTNS Efficacy for Detrusor Overactivity Treatment in MS Patients

The objectives of PTNS introduction originally in patients suffering from OAB were to activate the tibial nerve and enhance bladder control. Research has indicated that PTNS may be a beneficial OAB treatment. Randomized controlled studies show that PTNS has a success rate that is significantly higher than placebo. Compared to control groups, PTNS significantly improved bladder control and reduced urinary urgency and frequency in systematic reviews and meta-analyses [27]. The findings affirm the positive efficacy of PTNS as a minimally invasive, non-surgical, and non-hormonal treatment for OAB. Furthermore, it has been demonstrated that PTNS has a long-lasting impact: patients report symptom improvement for one to three months following the procedure [27].

Periodic stimulations to maintain the therapeutic effects have also been used to illustrate the durability of the improvement achieved by PTNS [28]. PTNS is safe, and no significant side effects have been documented in the literature. Given these possibilities, PTNS might be made available early in OAB treatment. More recent meta-analysis findings also indicate that PTNS can positively impact daytime frequency, nocturia, urgency episodes, voided volume, and urge incontinence. These findings are well supported by Wang et al. (2020) [29], who reviewed 28 studies and 2,461 patients to conclude that PTNS is effective and safe in treating OAB symptoms, with the most significant complication being pain at the puncture site, albeit with a very low incidence.

However, data for progressive neurologic conditions like MS remain limited, with only a handful of studies showcasing the efficacy of such treatments in MS patients.

A multicenter, prospective trial involving 21 MS patients (5 men and 16 women) experiencing LUTS unresponsive to anticholinergics who underwent 12 sessions of PTNS. The study demonstrated a notable reduction in daytime frequency, nocturia, and mean post-micturition residual volume. The mean voided volume increased from 182 ± 50 mL to 225 ± 50 mL (*p* = 0.003). Overall, 89% of patients reported a treatment satisfaction level of at least 70% and a significant improvement in QoL questionnaires. The patients did not report adverse events [21].

According to Marzouk et al. [30], who investigated the therapeutic efficacy of PTNS in addressing neurogenic OAB (NOAB) symptoms in males with MS, PTNS represents a promising and potentially beneficial treatment option for alleviating the symptoms. The trial included 40 remitting–relapsing males with MS experiencing moderate NOAB symptoms, randomly assigned to either a control group treated with a selected therapeutic exercise program for strengthening pelvic floor muscles or an intervention group receiving additional PTNS. Results demonstrated a significant improvement in all voiding parameters compared to baseline and the control group, with the PTNS group showing a substantial decrease in post-treatment mean episodes of nighttime frequency, urgency, and urgency incontinence. Additionally, there was a significant reduction in post-treatment overactive bladder symptoms score (OVBS score) in the PTNS group compared to the control group. Urodynamic parameters, including bladder capacity and compliance, detrusor overactivity, maximum flow rate, and post-void residual volume, significantly improved in the electrostimulation group compared to the control group [30].

PTNS administered over a 12-week period was found to enhance urodynamic parameters in a study involving 19 MS patients with DO [31]. The findings revealed an increase in mean urine volume, elevated maximal cystometric capacity, and suppressed detrusor contraction. Furthermore, PTNS has demonstrated efficacy in ameliorating LUTS in individuals with MS [31].

### 5.2. Long-Term PTNS Effects and Combination Treatments

Zecca et al. reported that MS patients after PTNS treatment continue to present after 24 months’ (long-term follow-up) stable and sustained improvement in DO symptoms [32]. In this study, there was a substantial reduction in nocturia, from four to two episodes after the treatment (*p* = 0.0010).

A study involving 21 MS patients investigated the sustained therapeutic effects of PTNS over the long term. The study implemented a tapering protocol of PTNS treatment over 6, 9, and 12 months. Throughout the duration of the study, significant improvements were observed in all voiding diary parameters at the 6th, 9th, and 12th months. Comparisons of mean values between baseline and the 12-month parameters indicated a reduction in daytime frequency of 5.4 voids daily, a decrease in urge incontinence of 3.4 episodes daily, a decline in urgency episodes of 7.4 episodes daily, a decrease in nocturia of 2.6 voids, and an improvement in voided volume of a mean of 72.1 mL [33].

In 2020, a retrospective study spanning 18 months assessed 74 patients, including 19 with MS and neurogenic OAB who underwent a 12-week PTNS treatment. While the entire neurogenic cohort exhibited partial improvements in frequency and incontinence episodes, these changes were not statistically significant (*p* = 0.3 and *p* = 0.8, respectively). However, OAB symptoms, as measured by the International Consultation on Incontinence Questionnaire—Overactive Bladder (ICIQ-OAB), showed a significant reduction (*p* = 0.04), and quality of life (QOL), assessed by ICIQ-LUTS QoL, improved (*p* = 0.05). Notably, MS patients were more likely to opt for top-up maintenance treatment compared to other neurological patients and the idiopathic group [34].

Andersen et al. [35] conducted a study over four years in a daily outpatient clinic to evaluate the clinical effectiveness of PTNS for idiopathic and neurogenic OAB. After a 12-week PTNS regimen, patients received monthly maintenance treatment. The results revealed a significant positive impact on patient-reported outcome measures, bladder diary, and ICIQ-OAB scores. Remarkably, 62% of patients continued maintenance PTNS, indicating sustained efficacy. PTNS demonstrated equal effectiveness for men and women in both idiopathic and neurogenic OAB subgroups, advocating for its consideration as a standard treatment option in urological departments.

According to Majdinasab et al. [36], solifenacin sulfate (SS) may offer advantages over PTNS in managing OAB symptoms in individuals with MS. Both SS and PTNS have shown efficacy in ameliorating OAB symptoms among MS patients. Nevertheless, patients have reported a more favorable experience with SS, particularly regarding daytime frequency, urinary incontinence, and overall treatment satisfaction. Some health-care providers may consider augmenting PTNS therapy with SS to enhance patient outcomes. Overall, the combination of SS plus PTNS appears to outperform either intervention used in isolation in specific patient subpopulations [37].

### 5.3. PTNS Prognostic Indicators and Alternative Approaches

Limited information exists regarding prognostic indicators for the success or failure of PTNS. Previous research has suggested that OAB patients who exhibit exceptionally low baseline cystometric capacity are more likely to be non-responders to PTNS treatment [38]. Additionally, individuals with a low SF-36 Mental Component Summary were found to be at higher risk of treatment failure. Despite comparable disease severity to patients with better mental health, these individuals exhibited poorer scores on disease-specific quality-of-life assessments. Thus, patients experiencing mental health challenges may not be optimal candidates for PTNS therapy. The notable effectiveness of PTNS, coupled with its favorable patient tolerance, underscores the advantages of employing this treatment either independently or alongside other rehabilitation programs to address neurogenic bladder dysfunction in MS patients [21,31,39,40].

An alternative, less invasive approach involving transcutaneous tibial nerve stimulation (TTNS) using surface electrodes may offer therapeutic benefits for individuals with MS experiencing DO, as opposed to PTNS. A study involving 70 patients demonstrated the efficacy of daily 20 min TTNS sessions over a 3-month period. The results indicated a reduction in urinary urgency (82% of patients), frequency (66.7% of patients), and incontinence (62% of patients) [41]. Studies conducted on non-MS patient cohorts indicate comparable efficacy between percutaneous PTNS and transcutaneous PTNS in managing OAB and bowel dysfunction [42,43]. Recently, a novel self-adhesive ambulatory device called GEKO (Firstkind, Buckinghamshire, UK) was introduced. The GEKO system stimulates the tibial nerve transcutaneously, enabling patients to move about freely while receiving treatment [44].

There is a scarcity of data concerning PTNS efficacy in managing DU and DSD. Given the limited evidence available, we are unable to recommend these methods for MS patients. Further research is warranted to ascertain their potential benefits and safety profile in this population.

## 6. Financial Aspects of PTNS in Modern Health-Care Systems

Numerous studies have explored the financial aspects of treatments for OAB, particularly focusing on PTNS and SNS. Due to differences in health-care systems across countries, the reported costs vary. For instance, Kurdoglu et al. [45] reported that third-line PTNS in Texas reduced the costs of medications, physician and nurse visits in OAB patients. Despite the financial advantages, PTNS was not commonly utilized among patients undergoing third-line treatment for OAB. Its usage was predominantly observed in urban settings and among older individuals, as well as those enrolled in cost-conscious health maintenance organizations. Staskin [46] calculated the financial costs of PTNS to SNS for OAB syndrome in the USA, revealing PTNS to be the more economical option with a cost of 7565 USD for a three-year treatment compared to SNS, which costs 24,681 USD for the same duration. Chen et al. estimated the cost of one year of PTNS treatment to be 4375 USD per patient [47]. Martinson et al. [48] also concluded that PTNS incurred substantially lower costs compared to SNS in the USA. Despite variations among countries, it appears that PTNS generally offers a more cost-effective solution compared to SNS.

One of the financial advantages of PTNS is that health-care professionals can perform these procedures in community-based urology practices. Sirls et al. [49] reported that patients in an office-based setting presented a decrease in OAB symptoms after weekly PTNS. Long-term feasibility might be restricted due to nonadherence to maintenance and insufficient efficacy. Factors such as copay, travel distance and employment status did not correlate with noncompliance.

## 7. Anticipating Future Enhancements and Evolutions in PTNS Therapy

### 7.1. Is There Any Place for Conditional Stimulation?

Inhibition of detrusor contraction is necessary in the event of an involuntary contraction. PTNS could be introduced when intravesical pressure begins to rise and ceased when pressure returns to normal levels. This approach is termed conditional or event-triggered stimulation.

Fjorback et al. [50] investigated the acute urodynamic impacts of electrical stimulation of the posterior tibial nerve in a specific subset of MS patients. The stimulation was applied conditionally to the posterior tibial nerve when detrusor pressure exceeded 10 cm H_2_O. However, PTNS failed to suppress detrusor contraction, and there was no observed reduction in urgency during stimulation.

Conversely, Amarenco et al. [51] proposed that PTNS yields measurable acute effects on urodynamic parameters. The observed reduction in bladder overactivity provides compelling support for considering PTNS as a viable non-invasive treatment option in clinical settings. Similarly, Kabay et al. [39] explored the acute impact of unilateral PTNS on bladder function, aligning with the findings of the aforementioned study. However, their investigation involved two successive bladder fillings in MS patients, presenting a limitation in their methodology.

### 7.2. Implantable Devices for PTNS in MS Patients

The BlueWind RENOVA device represents an innovative remote tibial nerve stimulation technology designed for convenient use without the need for batteries. Comprising an implant, an external control unit (ECU), and a physician programmer, this system offers a comprehensive approach to treatment [52]. The BlueWind RENOVA operates on a closed-loop mechanism, allowing patients to wear the ECU solely during treatment sessions while maintaining the freedom to move unrestrictedly in between. Initial trials reported a response rate of 71% over a six-month follow-up period [53]. Long-term data spanning three years revealed that approximately 75% of patients remained responders after 36 months of system activation [54]. The BlueWind RENOVA represents an implantable device known to maintain stable energy delivery from the ECU to the implant throughout treatment sessions characterized by a reduced migration risk. However, the requirement for open surgery for implantation represents a notable drawback compared to percutaneous PTNS.

Another recent advancement comes in the form of a battery-free implantable device (StimRouter™, Bioness Inc., Valencia, CA, USA), designed for tibial nerve stimulation. The device can be easily positioned with local anesthesia and consists of a personalized lead powered transcutaneously by an external pulse generator. Its components include a receiver, electrodes, and an anchoring system within the implanted lead [55]. During the implantation process, ultrasound or fluoroscopic imaging may be utilized to guide the placement of stimulation probes, which are employed to assess stimulation and ensure accurate targeting of the peripheral nerve. In June 2019, the inaugural MS patient with OAB and urge incontinence received the implant. The outcome revealed a reduction in urge incontinence, urgency episodes, and frequency. Subsequently, a prospective, single-center clinical trial involving MS patients treated with self-administered PTNS delivered by a StimRouter over a 24-week period was conducted. According to the study, the StimRouter represents a minimally invasive, magnetic resonance imaging-compatible, self-administered neuromodulation device that fosters both objective and subjective improvements in OAB symptoms and related QoL among MS patients with refractory LUTS [56].

The eCoin, developed by Valencia Technologies Corporation, represents a novel neuromodulation technology approved by the FDA, showing promising initial results [57,58]. However, there are limited available data concerning its application in MS patients. Analysis of its effectiveness indicates that 68% of patients with refractory OAB experienced a 50% reduction in UUI episodes 48 weeks post-activation, with 16% of those implanted reporting device-related events within 52 weeks of implantation [59]. The advantages of this device are the simple implantation technique and the battery-powered system that eliminates the need for patient involvement in its function, thereby promoting high adherence. Nevertheless, a drawback of this approach is the inability of patients to adjust the frequency of therapy sessions based on evolving symptoms. During maintenance therapy, treatment sessions are automatically scheduled every 15 days, potentially occurring at times when the patient may not desire treatment. Additionally, concerns include the risk of migration and the necessity for battery replacement, which entails additional procedures for patients.

### 7.3. Future Perspectives

The majority of PTNS research has adhered to a standardized protocol: 10 to 12 weekly sessions lasting 30 min each, with preset and relatively fixed stimulation parameters and the insertion of only one needle at a time. However, altering the treatment regimen or stimulation parameters could potentially yield different, potentially improved outcomes. This includes exploring bilateral therapy instead of unilateral. An accelerated stimulation schedule offers the advantage of achieving clinical results more rapidly. In terms of stimulation parameters, there is general consensus that pulse intensity should be set to a well-tolerated level, with studies suggesting that frequencies below 20 Hz may yield better results, particularly frequencies as low as 5–6 Hz. Similarly, adjustments in pulse duration, where the standard setting for PTNS is 0.2 milliseconds, could also impact outcomes.

## 8. PTNS Side Effects and Limitations

PTNS has emerged as a promising treatment option for various lower urinary tract symptoms, with reported side effects generally being mild and well-tolerated by patients. Common complications primarily include mild discomfort or pain at the site of needle insertion and mild leg cramps [30,33].

Despite these minor drawbacks, PTNS is increasingly recognized for its efficacy, safety, and lack of associated side effects. However, patient selection plays a crucial role in achieving optimal therapeutic outcomes, with emphasis placed on factors such as compliance, autonomy, and cognitive abilities [7]. Despite its apparent effectiveness in alleviating bothersome symptoms like urgency in over 80% of treated patients, caution is warranted due to the limited sample size and potential bias risk in the referenced studies [21]. Furthermore, there is limited evidence for the effectiveness of PTNS in the male population [6,21].

Ongoing research efforts are dedicated to enhancing the quality of implantable devices for tibial nerve stimulation, yielding promising results. This innovative approach not only proves to be well tolerated and safe but also addresses the logistical challenges associated with maintenance therapy, potentially reducing the need for frequent hospital visits. Therefore, while PTNS holds significant promise, further research is essential to comprehensively evaluate its efficacy and safety profile [7].

## 9. Conclusions

In wrapping up, the pervasive issue of bladder dysfunction significantly affects the life quality of nearly 80% of individuals living with MS, highlighting the need for alternative therapeutic strategies beyond conventional methods. This narrative review brings to light the potential of PTNS as an innovative solution for improving bladder control and overall life satisfaction among the MS community. The compilation of various study findings presents PTNS as an effective measure, particularly when the peripheral tibial nerve is stimulated for 20 min daily across a three-month span, yielding consistent improvements and high acceptance among patients. Notably, while some research indicates the absence of immediate benefits from electrical stimulation of the posterior tibial nerve, extended PTNS sessions have been linked to ongoing enhancements in symptoms related to the lower urinary tract. The significant effectiveness and patient receptiveness towards PTNS highlight its viability as either an independent or supplementary therapy for managing neurogenic bladder issues in MS sufferers.

Despite these positive outcomes, the call for additional, varied research remains, especially to understand its effects on specific groups like men with idiopathic OAB. The evidence suggests that PTNS could offer relief to individuals with OAB syndrome unresponsive to conventional treatments, further supported by its commendable safety profile, with negligible adverse effects reported. Nonetheless, the adoption of PTNS should be viewed as an adjunct rather than a cure-all for bladder issues within the MS demographic. The choice to implement PTNS ought to stem from an in-depth consultation with medical practitioners, meticulously balancing its advantages against possible constraints and tailored to the unique circumstances of each patient, including the severity of symptoms and overall health condition. As ongoing research progresses, PTNS is poised to become a key player in the tailored management of bladder dysfunction in MS, promising to elevate patient care standards and personalize treatment pathways.

## Figures and Tables

**Table 1:** PTNS possible mechanisms and clinical effects.

Possible Mechanism	Clinical Effects
Impact of PTNS treatment on long-latency somatosensory evoked potentials (LL-SEPs)	Alterations in brain activity following PTNS relieving detrusor overactivity
PTNS effect on sacral and suprasacral centers involved in stimulus processing, potentially engaging relevant brain cortical regions.	Suppress detrusor activity by depolarizing somatic sacral and lumbar afferent fibers.
Influence on spinal cord function	Reduction in C-fos activity (a marker of neuronal metabolism)
Reduced mast cell population in the bladder	Reduced marker of cystitis-associated lower urinary tract dysfunction

PTNS: posterior tibial nerve stimulation.

## Data Availability

The datasets used and/or analyzed during the current study are available from the corresponding author on reasonable request.
